# *Cystoisospora suis* Control in Europe Is Not Always Effective

**DOI:** 10.3389/fvets.2020.00113

**Published:** 2020-03-04

**Authors:** Barbara Hinney, Vojislav Cvjetković, David Espigares, Jonas Vanhara, Christoph Waehner, Bärbel Ruttkowski, Radinka Selista, Daniel Sperling, Anja Joachim

**Affiliations:** ^1^Department of Pathobiology, Institute of Parasitology, University of Veterinary Medicine Vienna, Vienna, Austria; ^2^CEVA Santé Animale, Libourne, France

**Keywords:** *Isospora suis*, toltrazuril, coccidiosis, piglets, swine, diarrhea, disinfection

## Abstract

After introduction of the anticoccidial toltrazuril for the metaphylactic treatment of suckling piglet coccidiosis, only few field evaluations on the effect of treatment against the causative agent, *Cystoisospora suis*, were performed. In 2018, a field study was conducted to detect the presence of the parasite on pig farms in four different European countries, and to evaluate management parameters possibly associated with infection and disease. A total of 49 farms from Austria, the Czech Republic, Germany and Spain were included. Repeated pooled fecal samples from 603 litters were taken in the 2nd and 3rd week of life. Samples were examined by autofluorescence for the presence of *C. suis*, and fecal consistency was scored. For each farm a questionnaire was provided to document management and treatment history. Feces scored as diarrhoeic were not significantly more often positive for *C. suis* than non-diarrhoeic feces but samples from litters with previously reported occurrence of diarrhea were significantly more often positive (*p* = 0.000). Pasty feces were significantly more often positive than those of other consistency (*p* = 0.005). Overall, 71.4% of the farms and 50.1% of the litters were positive for *C. suis* at least once. The prevalence on the farms reached up to 100%. Diarrhea was seen in samples from 53.1% of the farms (9.6% of the litters). *Cystoisospora suis* was diagnosed on 80.8% of the farms with vs. 60.8% of those without diarrhea. Toltrazuril was applied on 30 farms, and of these 53.3% had diarrhoeic samples and 66.7% were positive for *C. suis* vs. 19 farms that did not use toltrazuril with 52.6% diarrhoeic and 79.0% *C. suis* positive samples (*p* > 0.05). Only on two farms a disinfectant with activity against coccidia was used, and *C. suis* was not detected there. Current control of *C. suis* appears to be insufficient on the majority of the examined farms. These findings highlight the importance of correct application of medication, and an effective hygiene management. To maintain effective parasite control, efficacy monitoring of the control measures should be implemented.

## Introduction

*Cystoisospora suis* (*C. suis*; formerly *Isospora suis*) is a common pathogen in suckling piglets worldwide with high prevalence rates reported previously, e.g., 76% of farms in Germany, Austria and Switzerland (1); 67% in Poland (2); 58% in Sweden (3); 70% in Canada (4); 82% in Venezuela (5); and 66.3% in China (6).

Piglets become infected by oral uptake of sporulated oocysts. Sporozoites are released from these oocysts and penetrate epithelial cells of the small intestine, replicate, and progressively destroy intestinal cells. This leads to intestinal lesions including villous necrosis, atrophy and fusion, and frequently results in non-haemorrhagic diarrhea. Generally, morbidity is high while mortality is low (7). However, piglet health can significantly deteriorate by bacterial or viral co-infections (8–10). As piglets that suffered from cystoisosporosis often show failure to thrive, the infection can result in marked production losses (11–13). The disease tends to take a more serious course in very young piglets while age resistance results in mostly subclinical infections in weaned animals [as reviewed in (14)]. To prevent early exposure of piglets to the parasite, reduction or inactivation of infectious oocysts is key to the control of cystoisosporosis. Efficient hygiene strategies include steam-cleaning and the application of a disinfectant with anticoccidial efficacy (15). On pig farms where *Cystoisospora*-related diarrhea occurs, metaphylactic treatment with the coccidiocidal drug toltrazuril is recommended. It has been shown to be efficient in several laboratory and field studies and can thus enhance animal welfare as well as farm productivity (13, 16–18). Toltrazuril is now frequently used to control piglet cystoisosporosis in Europe (19). As *C. suis* induces intestinal damage during prepatency, i.e., before oocyst are excreted with the feces, efficient treatment has to be applied to piglets exposed to infection before the parasite can be diagnosed coproscopically (16, 17). This highlights the importance of correct diagnosis of the presence of *C. suis* in a herd in order to obtain relevant information on the indication for and the correct time point of treatment (20).

With this study we aimed to investigate the occurrence of *C. suis* on selected conventional pig breeding farms in four European countries and to evaluate management and treatment strategies for their possible association with *C. suis* and cystoisosporosis. We hypothesized that toltrazuril application and/or appropriate hygiene management strategies reduce *C. suis* infections in litters compared to farms that did not perform one or both of these strategies. Also we hypothesized that diarrhea occurred more often on farms where *C. suis* was detected.

## Materials and Methods

### Farms and Samples

A non-randomized cross-sectional field study was performed. The aim was to evaluate infection rates on swine farms with different management conditions in four European countries. With this we wanted to receive an updated insight in possible influencing variables. Of particular interest was the effect of toltrazuril treatment.

In 2018, we investigated 49 farms from four different countries, 7 from Austria, 17 from the Czech Republic, 7 from Germany and 18 from Spain. These farms were selected arbitrarily. From each farm, pooled litter samples (four samples/ litter collected from the floor) were obtained. We aimed to sample at least 10% of the litters that were born during the examination period, but a maximum of 30 litters/farms. To increase sensitivity each litter was sampled twice in one-week intervals due to the short periods of oocyst excretions (20). Number of litters examined/farm are shown in Supplementary Table 1. In 19 farms this minimum number of litters could not be sampled, in eight of these all litters were negative. As on these eight farms infection might have been missed, they were excluded from statistical analysis. In total 6–63 litters per farm were included.

### Coproscopical Analysis

Each sample was mixed well and aliquots of ca. 0.1–0.2 g of feces were examined by autofluorescence as described before (20). Samples were considered positive when at least one oocyst could be detected. Differentiation between *Cystoisospora suis* and *Eimeria* spp. was based on morphological features and confirmed by sporulation of positive samples (7). By repeated sampling we estimated to achieve a sensitivity of 80% and a specificity of 95%. Depending on the density of oocysts in a sample semi-quantitative scoring of the positive samples (from 1 = low grade to 3 = high grade) was included. In the laboratory of the Institute of Parasitology, fecal consistency was scored from 1 to 4 as described (21) and fecal scores 3 and 4 were considered as diarrhea.

For each farm a questionnaire was provided for information on farm structure, management, and medication of piglets during the suckling period as well as previous records of diarrhea (Data sheet 1). This questionnaire was completed by the farm-veterinarian. Questions and response rates are shown in Table 1.

**Table 1:** Topics addressed in the farm questionnaire and analyzed with regard to infections with *Cystoisospora suis* and diarrhea (dependent variables).

**Farm structure and management (independent variables)**	**Number of farms *C. suis* positive/ answer was “yes”**	***p*-values**	**Number of farms with diarrhea/answer was “yes”**	***p*-values**
• Number of sows [49, 41]	n.a.	0.328*	n.a.	0.773*
• Solely nursery farms? (yes/no) [49, 41]	14/17	0.646	9/17	0.938
• Number of other pigs [47, 39]	n.a.	0.472*	n.a.	0.863*
• Closed system (yes/no) [44, 36]	13/18	0.074	9/18	0.738
• All in all out? (yes/no) [48, 40]	30/3	0.738	18/34	0.533
**Health observations**				
• Diarrhea (yes/no) on farm level [43, 41]	21/22	0.124 **(0.049)**	n.a.	n.a.
• Diarrhea (yes/no) on litter level (percentage of litters with diarrhea on farms) [24,22]	n.a.	0.051*	n.a.	n.a.
**Treatment in the first 32 days of life**				
• Use of toltrazuril (yes/no)? [46, 41]	20/24	0.662	14/24	0.476
• Age of piglets at toltrazuril application [26, 22]	n.a.	n.a.	n.a.	n.a.
• Day of iron application? [48, 40]	n.a.	n.a.	n.a.	n.a.
• Use of antibiotics (yes/no), if yes which compound? [46, 38]	16/20	0,453	9/20	0.321
**Cleaning/disinfection measures**				
• Cleaning and disinfection of stables? (yes/no) [47, 41]	34/40	0.675	21/40	0.347
• Which disinfectant is used? [37, 31]	n.a.	n.a.	n.a.	n.a.

In square brackets: number of questionnaires (out of 49) that supplied information on this point, followed by the number of farms that could be included into statistical analysis. P-values were calculated with Pearson's χ^2^ test or the Mann–Whitney-U test (marked with an asterisk*).

*Bold value indicate the significant when farms with insufficient sample size were excluded*.

### Statistical Analysis

As management parameters were collected per farm, most analyses were performed on farm level. An association between diarrhea and *C. suis* infection was also analyzed on the level of litters (*n* = 603). Fecal score and C. suis-oocyst shedding were compared for all samples (*n* = 1,206). Testing for significance was done on a 95% CI with Pearson's x^2^ test (nominal data) and the Mann–Whitney-U test (metric data). All statistical analyses were performed using IBM SPSS Statistics 24 (IBM GmbH, Ehningen, Germany).

## Results

### Composition of Sampled Farms

All herds included in this study were from conventional pig farms with 65–10,000 sows/farm. In Austria, only farms with a maximum of 200 sows were examined, while in the other countries also farms with more than 2,000 sows were included (Table 2).

**Table 2:** Number of farms and litters positive for *C. suis* and diarrhea.

	**Country [N farms]**				
	**Austria *N* = 7**	**Czech Republic *N* = 17**	**Germany *N* = 7**	**Spain *N* = 18**	**Total *N* = 49**
Min-max number of sows/farm	65–200	250–2,600	95–6,500	240–10,000	65–10,000 Mean: 1249.7 Median: 600
N positive farms [%; 95% confidence interval]	5 [71.4;35.89–91.78]	12 [70.6;46.87–86.72]	3 [42.9; 15.82–74.95]	15 [83.3; 60.78–94.16]	35 [71.4; 57.59–82.15]
N farms >50 % positive litters [%]	0 [0.0]	3 [17.6]	1 [14.3]	12 [66.7]	16 [32.7]
N litters	78	161	71	293	603
N positive litters [%; 95% confidence interval]	9 [11.5; 6.19–20.50]	53 [32.9; 26.13–40.50]	11 [15.5; 8.88–25.65]	229 [78.2; 73.08–82.51]	302 [50.1; 46.10–54.06]
N farms with diarrhea [%]	4 [57.0]	8 [47.1]	4 [57.1]	10 [55.6]	26 [53.1]
N litters with diarrhea [%]	11 [14.1]	12 [7.5]	7 [9.9]	28 [9.6]	58 [9.6]

**Figure 1:**
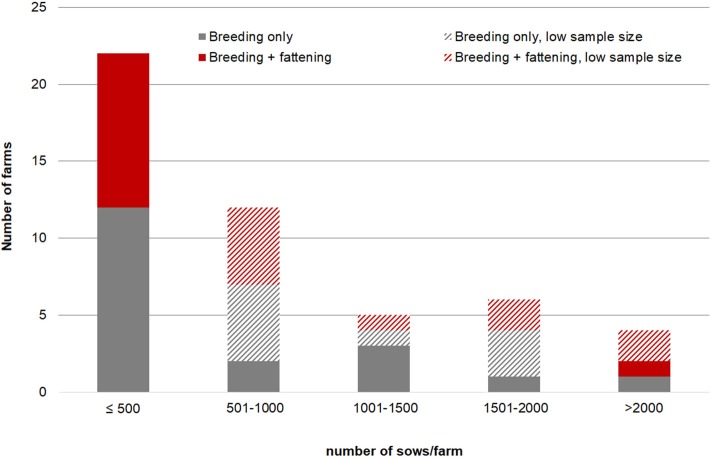
Number of nursery farms vs. farms with nursery and fattening, grouped according to number of sows.

Not all of those farms answered the questionnaire regarding their farming system (Table 1). Of the 49 farms 28 (57.1%) were pure nursery farms. The remaining farms also kept fatteners, and this was the case for 28.6% (Austria), 64.7% (Czech Republic), 57.1% (Germany) and 22.2% (Spain), respectively. With regard to the numbers of sows per farm, medium sized farms (1,001–1,500 sows) most often only had nurseries (Figure 1).

### Samples

As 603 litters were examined twice a total of 1,206 fecal samples were available, 39.3% of which were positive for *C. suis*; 15.0, 11.8, and 12.5% showed a low-, medium-, and high-grade excretion, respectively.

Diarrhea (fecal score 3 or 4) was observed in 5.1% (4.8% semi-liquid, 0.3% with liquid consistency), while 48.0% of fecal samples were firm and 46.8% pasty.

When comparing fecal scores in relation to the grade of *C. suis* infection, samples with pasty feces had a significantly higher grade for *C. suis* (*P* = 0.005), which was not the case for semi-liquid or liquid (diarrhoeic) feces.

### Occurrence of *C. suis* and Diarrhea on Litters and Farms

Overall, we examined 603 litters from 49 farms (mean 12.3 litters/farm) in the 2nd and 3rd week of life. A litter was considered coccidia/diarrhea-positive when it was positive at least in one out of the two samplings. For the further analysis no distinction was made between litters tested positive once or twice. In total 35 farms were positive for *C. suis* but only 23 were positive in both samplings (Supplementary Table 1). Thus, 24.5% of farms were identified as *C. suis*-positive which would have been classified as negative with a single sampling. The total number of litters that were tested positive for *C. suis* only varied slightly (43.6 vs. 45.8%) between both samplings, however, these were different litters so that the difference between sampling once and sampling twice differed significantly (*p* = 0.000).

**Figure 2:**
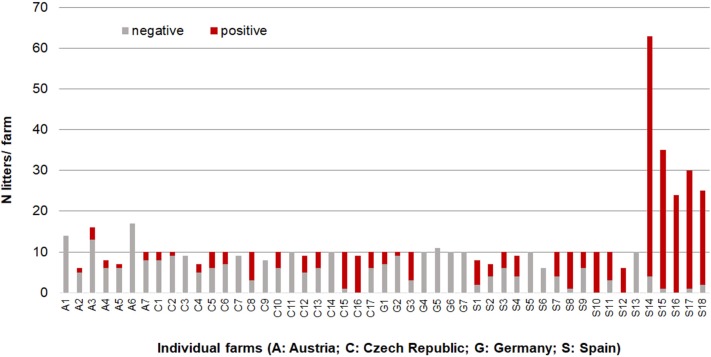
Fecal score in relation to excretion intensity with *Cystoisospora suis*.

A farm was considered positive when at least one litter was positive as defined above. Overall, 71.4% of the farms and 50.1% of the litters were positive for *C. suis* at least once (Supplementary Table 1). The prevalence on the farms varied greatly from 0.0 to 100%. Number of litters tested per farm and the proportion of *C. suis* positive litters are shown in Figure 2.

Anamnestic information on diarrhea was available for 359 litters from 24 farms, and 31.8% of these farms reported diarrhea. Of the litters with an anamnesis of diarrhea, 82.6% were positive for *C. suis*. This rate was significantly higher (*P* = 0.000) than that for litters without anamnestic diarrhea (58.1%).

Diarrhea was detected by scoring of the fecal samples in 9.6% of the litters and 53.1% of the farms (Table 2). Farm prevalences for diarrhea ranged between 0.0 and 80.0%. Of the litters classified as diarrhoeic (*n* = 58), 53.4% were positive for *C. suis*, while of the non-diarrhoeic litters (*n* = 545) 49.7% were positive. On the farm level, 80.8% of the farms with diarrhea-positive litters and 60.8% of the farms without diarrhoeic litters were positive for *C. suis*. When eight farms with an insufficient sample size negative for *C. suis* were excluded (see Material and Methods), farms where diarrhea was present had significantly more often an infection with *C. suis* (*P* = 0.049). These differences were, however, not significant when all farms were included (*P* = 0.124).

In the questionnaire, 26 of 43 farms reported a general problem with diarrhea. Of these 80.8% were *C. suis*-positive, compared to 17 farms with no problems with diarrhea of which 64.7% were *C. suis*-positive (*P* > 0.05).

### Occurrence of *C. suis* by Country

Farms from Germany had the lowest farm-related rate for *C. suis* infection, while the highest was observed in Spain (*P* > 0.05) (Table 2). Farms from Spain were also those with the highest on-farm-prevalence; on two thirds of these farms more than half of the litters were positive for *C. suis*, compared to less than one fifth in the other countries (Table 2). Compared to the on-farm prevalence of other countries, farms from Spain had significantly higher prevalence rates than the other countries (Austria: *P* = 0.006 for all farms, *P* = 0.003 when farms with low sample size were excluded; Czech Republic: *P* = 0.011/*P* = 0.003; Germany: *P* = 0.009/*P* = 0.003).

### Farm Size and Occurrence of *C. suis*

**Figure 3:**
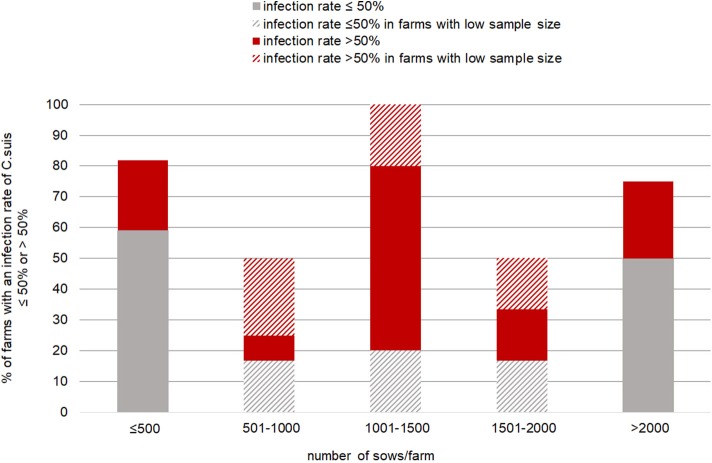
*Cystoisospora suis*-positive and negative litters (by autofluorescence) per individual farm.

Regarding the occurrence of *C. suis* on farms in relation to farms size, infection rates were highest on medium sized farms (1,001–1,500 sows), and this group of farms also had the highest on-farm-prevalence (Figure 3). These differences, however, were not significant (*P* > 0.05).

### Toltrazuril Treatment

#### Farm Level

Thirty farms administered toltrazuril to their piglets according to the questionnaire. On 66.7% of these farms at least one litter was positive for *C. suis* and on 53.3% diarrhea was detected. In the 19 farms that did not use toltrazuril, *C. suis* and diarrhea were present in 79.0 and 52.6%, respectively (*P* > 0.05). Twenty-six farms provided information about the age of the piglets upon toltrazuril application. For several farms, a range of time points was listed. Nine farms stated to treat piglets with toltrazuril not later than the third day of life (regarded as “early treatment”), while 10 farms treated after the third day of life (“late treatment”). Seven farms treated in an overlapping range between early and late time points. Significantly more farms applying “late treatment” (90.0%) compared to those treating early (44.4%) were positive for oocyst excretion (*P* = 0.033). This difference was even more obvious when the farms with insufficient sample sizes that were negative for *C. suis* were excluded (*n* = 4 farms for this variable, two with “early,” one with “late” treatment, one with overlapping time points). All nine farms with “late treatment” were *C. suis*-positive, while 57.1% of the seven farms with “early treatment” were *C. suis*-positive (*P* = 0.029).

#### Litter Level

Oocysts were detected in 52.5% (*n* = 200) of the litters from farms that stated to use toltrazuril and 46.0% (*n* = 102) of the litters from farms that did not apply toltrazuril. Diarrhea was present in 10.5% of litters (*n* = 40) and 8.1% (*n* = 18) in litters from farms without toltrazuril treatment. None of these differences were significant on the farm or litter levels (*P* > 0.05).

### Disinfection

Except for two (4.2%), all participating farms stated to clean and disinfect the stable, and 80.9% of those gave information on the product they used. The spectrum of activity of the disinfectants was checked against a list of disinfectants approved by the German Veterinary Society (http://www.desinfektion-dvg.de/index.php?id=2150) for their effect on coccidia. Only two farms applied (cresol-based) disinfectants listed as effective against coccidia. On these farms no oocysts were detected.

## Discussion

With an occurrence of *C. suis* on 71% of farms and up to 100% positive litters per farm the prevalence of *C. suis* in the examined population was considerable. These rates are in line with a study from Sweden that showed a cumulative herd prevalence of 76% in piglets up to 4 weeks (3). However, a direct comparison of infection rates between studies must be interpreted with caution due to non-representative sampling and different diagnostic methods with different sensitivities (20, 21). As Meyer et al. (22) pointed out, individual samples from piglets from a litter pooled to a litter sample are preferable for examination. We here considered taking single samples from the floor for a pooled litter sample as equivalent. In line with the recommendation of Meyer et al. (22), double sampling of litters in a weekly interval significantly increased sensitivity in our study. A third sampling may have further increased sensitivity (22), however, three samplings at weekly interval were not feasible for this study. As samples were not taken individually from animals but collected from the floor, pooled sample composition differed between both samplings, which may have lowered sensitivity to a certain extent.

Although farms from Spain had the highest infection rates while German farms had the lowest, this observation has to be interpreted with care. Convenient samples were taken without an a priori sample size calculation. Spain was the country were most samples were taken, so the probability to detect positive farms in this country was higher. The confidence intervals also clearly showed overlaps in infection rates between countries. In addition, different farm structures and sizes may have introduced a bias. True differences in infection rates between countries thus have to be confirmed by representative and stratified sampling. Interestingly, medium sized farms were those with the highest infection rates. With the data we collected with our questionnaire we could not detect any explanatory factors for this observation. The finding that medium-sized farms were mostly located in Spain might also mask the true reason for the higher infection rates in this sub-group. A further observation was that farms with fattening pigs had fewer *C. suis* infections than those that specialized in nurseries only. These again were mainly the middle-sized farms and the farms from Spain, so this observation could be related to farm size, country, or be confounded by other unknown factors and cannot be interpreted unambiguously.

Although oocyst shedding can occur despite toltrazuril treatment in single cases (13, 16, 23) a surprising finding of this study was that farms that used toltrazuril did not show a significant decrease of *C. suis* infection or diarrhea compared to farms without treatment. This is in distinct contrast to several laboratory and field-studies that demonstrated the high efficacy of toltrazuril in the control of cystoisosporosis experimentally and in the field (13, 16–18, 24). Conceivable reasons for that could be application errors or antiparasitic resistance. Although recently a toltrazuril-resistant *C. suis* strain was isolated from The Netherlands (19), we consider geographically expanded (but to date unrecognized) toltrazuril-resistance to be an unlikely explanation. The Dutch resistant strain was isolated from a single farm and surrounding farms were not affected (Jansen, pers. comm. 2017), so an extensive spread of resistance appears highly speculative, especially under high biosafety conditions which many piglet producing operations are employing. Therefore, more likely reasons of failure to control *C. suis* must be considered first. These include significant application errors of toltrazuril and the lack of a sufficient hygiene management. Application of toltrazuril is only fully efficient when given regularly and timely and at the right dosage to all piglets at risk. Delayed or sporadic treatment cannot be expected to result in a significant benefit. Nonetheless the possibility of resistance should not be disregarded and further studies on the spread of resistance against anticoccidial compounds are necessary.

Interestingly, we could observe a significantly lower occurrence of *C. suis* in farms that applied toltrazuril not later than on the third day of life compared to those that treated after this time point. Although full efficacy of treatment up to the 5th day of life (2 days after infection) was confirmed for toltrazuril in experimental studies (17), earlier application [used by e.g., (9)] might be more effective under field conditions. Additionally, good hygiene management is essential for the control of cystoisosporosis. We could only identify two farms that stated to use disinfectants that are effective against coccidia; both were negative for *C suis*. Although these few cases cannot be statistically evaluated, the regular use of disinfectants with anticoccidial action is strongly recommended (15) to inactivate as many oocysts as possible for reduction of the infection pressure in the new-born piglets' environment.

While diarrhoeic fecal scores were not correlated with the detection of *C. suis* in a sample, pasty feces correlated significantly with the presence of oocysts, as did the anamnestic report of diarrhea in litters. One reason for the difference between detected and anamnestic diarrhea in our study could be that semi-liquid and liquid fecal matter is difficult to collect from the (especially slatted) floor so that formed to pasty feces might have been overrepresented in the sampling. This assumption is corroborated by the correlation between diarrhea and *C. suis* excretion on the farm level. Another reason for the poor correlation between a diarrhoeic fecal score and *C. suis* detection in samples may also be due to the finding that the peak of oocyst excretion often does not coincide with diarrhea, resulting in a poor correlation between oocyst excretion and fecal consistency (23). The finding that pasty feces was significantly correlated to *C. suis* infection supports this explanation. In addition, although *C. suis* is known to be a causative agent of piglet diarrhea, piglet scours in this study might also have been caused by a variety of other pathogens.

Based on our findings, we strongly recommend to monitor the effects of toltrazuril treatment on farms and to pursue regular diagnosis of the possible causes of piglet diarrhea and stunted growth to obtain maximum treatment benefits in terms of piglet health and performance.

With appropriate coproscopical examination (20) also toltrazuril-resistance could be detected early, and this would allow for a quick intervention to minimize its further spread. If toltrazuril is prescribed, the pig farmer should be informed about the correct application of the drug. Additionally, the correct application and efficiency of the drug should be monitored by the responsible veterinarian. Also, accompanying measures such as inactivation of infectious oocysts by suitable disinfectants are essential.

In conclusion, *C. suis* is still a common enteropathogen of suckling piglets in the investigated areas. Control does not seem to be sufficiently elaborated on several of the investigated farms. To reveal the explanatory factors a thorough analysis of farms, ideally as part of a cohort study, should be performed.

## Data Availability Statement

The datasets generated during and/or analysed during the current study are available from the corresponding author on reasonable request.

## Ethics Statement

For this study samples from living animals were collected from the floor during farm visits by the attending veterinarians (see authors' contributions), so that no animals were directly involved. No conditions in the examined farms were changed. Due to the character of an observational study without manipulation on animals, an ethical approval was not necessary. Farmers consented to the participation orally by filling out the questionnaire.

## Author Contributions

AJ and DS designed the study. VC, DE, JV, and CW organized the sample and data collection. BH, BR, and RS analyzed the samples. BH carried out the statistical analysis. AJ and BH drafted the manuscript. All authors have read and approved of the submitted version of the manuscript.

### Conflict of Interest

DS, VC, DE, JV, and CW are employees of Ceva. No member of the staff of the Vetmeduni Vienna involved in the trial received allowances or other personal benefits from the Sponsor. The remaining authors declare that the research was conducted in the absence of any commercial or financial relationships that could be construed as a potential conflict of interest.

## References

[1] Mundt HC Cohnen A Daugschies A Joachim A Prosl H Schmäschke R. Occurrence of isospora suis in Germany, Switzerland and Austria. J Vet Med B. (2005) 52:93–7. 10.1111/j.1439-0450.2005.00824.x 15752269

[2] Karamon J Ziomko I Cencek T. Prevalence of *Isospora suis* and *Eimeria* spp. in suckling piglets and sows in Poland. Vet Parasitol. (2007) 147:171–5. 10.1016/j.vetpar.2007.03.029 17467906 PMC7130805

[3] Pettersson E Hestad S Möttus I Skiöldebrand E Wallgren P Rotavirus and *Cystoisospora suis* in piglets during the suckling and early post weaning period, in systems with solid floors and age segregated rearing. Porcine Health Manag. (2019) 8:5–7. 10.1186/s40813-019-0114-0 PMC6368768 30788133

[4] Aliaga-Leyton A Webster E Friendship R Dewey C Vilaça K Peregrine AS. An observational study on the prevalence and impact of Isospora suis in suckling piglets in southwestern Ontario, and risk factors for shedding oocysts. Can Vet J. (2011) 52:184–88. 21532828 PMC3022462

[5] Pinilla León JC da Silva Borges N Epidemiological aspects of *Cystoisospora suis* in swine herds located at the Central region of Venezuela. Rev Mex de Cienc Pecuarias. (2018) 9:278–95. 10.22319/rmcp.v9i2.4478

[6] Zhang WJ Xu LH Liu YY Xiong BQ Zhang QL Li FC. Prevalence of coccidian infection in suckling piglets in China. Vet Parasitol. (2012) 190:51–5. 10.1016/j.vetpar.2012.05.015 22694832

[7] Joachim A Shrestha A Coccidiosis of pigs. In: Coccidiosis in Livestock, Poultry, Companion Animals, and Humans, eds Dubey J Boca Raton P. CRC Press (2020). p. 125–45. 10.1201/9780429294105-11

[8] Vitovec J Koudela B Kudweis M Stepanek J Smid B Dvorak R. Pathogenesis of experimental combined infections with *Isospora suis* and Rotavirus in conventional and gnotobiotic piglets. J Vet Med B. (1991) 38:215-26. 10.1111/j.1439-0450.1991.tb00864.x 1858460

[9] Mengel H Krüger M Krüger MU Westphal B Swidsinski A Schwarz S. Necrotic enteritis due to simultaneous infection with Isospora suis and clostridia in newborn piglets and its prevention by early treatment with toltrazuril. Parasitol Res. (2012) 110:1347–55. 10.1007/s00436-011-2633-8 21968954

[10] Matsubayashi M Takayama H Kusumoto M Murata M Uchiyama Y Kaji M. First report of molecular identification of *Cystoisospora suis* in piglets with lethal diarrhea in Japan. Acta Parasitol. (2016) 61:406–11. 10.1515/ap-2016-0054 27078667 PMC7088846

[11] Sanford SE Porcine neonatal coccidiosis: clinical, pathological, epidemiological and diagnostic features. Californ Vet. (1983) 37:26–30.

[12] Lindsay D Blagburn B Powe T Enteric coccidial infections and coccidiosis in swine. Compend Cont Educ Pract. (1992) 14:698–702.

[13] Kreiner T Worliczek HL Tichy A Joachim A. Influence of toltrazuril treatment on parasitological parameters and health performance of piglets in the field–an Austrian experience. Vet Parasitol. (2011) 183:14–20. 10.1016/j.vetpar.2011.07.019 21820246

[14] Worliczek HL Mundt HC Ruttkowski B Joachim A Age, not infection dose, determines the outcome of Isospora suis infections in suckling piglets. Parasitol Res. (2009) 1(Suppl. 105):157–62. 10.1007/s00436-009-1507-9 19575237

[15] Straberg E Daugschies A. Control of piglet coccidiosis by chemical disinfection with a cresol-based product (Neopredisan 135-1). Parasitol Res. (2007) 101:599–604. 10.1007/s00436-007-0521-z 17364163

[16] Mundt HC Mundt-Wüstenberg S Daugschies A Joachim A. Efficacy of various anticoccidials against experimental porcine neonatal isosporosis. Parasitol Res. (2007) 100:401–11. 10.1007/s00436-006-0314-9 17048000

[17] Joachim A Mundt HC. Efficacy of sulfonamides and Baycox® against Isospora suis in experimental infections of suckling piglets. Parasitol Res. (2011) 109:1653–59. 10.1007/s00436-011-2438-9 21556685

[18] Rypula K Porowski M Kaba J Gorczykowski M Deniz A. Effect of isosporiasis prevention with toltrazuril on long-term pig performance. Sci World J. (2012) 2012:486324. 10.1100/2012/486324 22547982 PMC3322557

[19] Shrestha A Freudenschuss B Jansen R Hinney B Ruttkowski B Joachim A. Experimentally confirmed toltrazuril resistance in a field isolate of *Cystoisospora suis*. Parasit Vect. (2017) 10:317. 10.1186/s13071-017-2257-7 28662668 PMC5492287

[20] Joachim A Ruttkowski B Sperling D. Detection of *Cystoisospora suis* in faeces of suckling piglets - when and how? A comparison of methods. Porcine Health Manag. (2018) 4:20. 10.1186/s40813-018-0097-2 30250747 PMC6145109

[21] Joachim A Altreuther G Bangoura B Charles S Daugschies A Hinney B. WAAVP guideline for evaluating the efficacy of anticoccidials in mammals (pigs, dogs, cattle, sheep). Vet Parasitol. (2018) 253:102–19. 10.1016/j.vetpar.2018.02.029 29604993

[22] Meyer C Joachim A Daugschies A. Occurrence of Isospora suis in larger piglet production units and on specialized piglet rearing farms. Vet Parasitol. (1999) 82:277–84. 10.1016/S0304-4017(99)00027-8 10384903

[23] Mundt HC Joachim A Becka M Daugschies A. *Isospora suis*: an experimental model for mammalian intestinal coccidiosis. Parasitol Res. (2006) 98:167–75. 10.1007/s00436-005-0030-x 16323027

[24] Maes D Vyt P Rabaeys P Gevaert D. Effects of toltrazuril on the growth of piglets in herds without clinical isosporosis. Vet J. (2007) 173:197–9. 10.1016/j.tvjl.2005.07.002 16122955

