# Supplementary material for: Cystoisospora suis Control in Europe Is Not Always Effective
Source: Front Vet Sci. 2020 Mar 4;7:113. doi: 10.3389/fvets.2020.00113 (PMC7064465; doi:10.3389/fvets.2020.00113)
Supplement: Supplementary file 1 [file Table_1.pdf]

Supplementary Table 1: Presentation of sampled farms, sampled litters and the number of positive litters. A = Austria, C = Czech Republic; G = Germany; S = Spain. * = fewer litters sampled than required but *C. suis* was detected; ** = fewer litters sampled than required and all were negative for *C. suis*, these farms were excluded from statistical analysis.

Farm	N sows	Estimated number of litters in examination period	N sampled litters	N litters positive only at one of the two samplings	N litters positive at both samplings	% of litters positive for *C. suis*
A1	160	27	14	0	0	0
A2	65	11	6	1	0	17
A3	200	33	16	0	3	19
A4	106	18	8	1	1	25
A5	85	14	7	1	0	14
A6	100	17	17	0	0	0
A7	98	16	10	0	2	20
C1	2600	433	10*	2	0	20
C2	480	80	10	0	1	10
C3	1650	275	9**	0	0	0
C4	400	67	7	2	0	29
C5	350	58	10	2	2	40
C6	300	50	10	3	0	30
C7	600	100	9**	0	0	0
C8	2000	333	10*	4	3	70
C9	700	117	8**	0	0	0
C10	700	117	10*	4	0	40
C11	540	90	10	0	0	0
C12	295	49	9	4	0	44
C13	500	83	10	4	0	40
C14	1000	167	10**	0	0	0
C15	1000	167	10*	2	7	90
C16	500	83	9	9	0	100
C17	250	42	10	1	3	40
G1	6500	1083	10*	3	0	30
G2	1200	200	10*	1	0	10
G3	1200	200	10*	6	1	70
G4	95	16	10	0	0	0
G5	1700	283	11**	0	0	0

Farm	N sows	Estimated number of litters in examination period	N sampled litters	N litters positive only at one of the two samplings	N litters positive at both samplings	% of litters positive for *C. suis*
G6	500	83	10	0	0	0
G7	1900	317	10**	0	0	0
S1	500	83	8	5	1	75
S2	240	40	7	3	0	43
S3	1250	208	10*	3	1	40
S4	300	50	9	3	2	56
S5	920	153	10**	0	0	0
S6	580	97	6**	0	0	0
S7	270	45	10	3	3	60
S8	650	108	10*	5	4	90
S9	2000	333	10*	0	4	40
S10	400	67	10	2	8	100
S11	600	100	10	6	1	70
S12	600	100	6*	3	3	100
S13	10000	1667	10	0	0	0
S14	9500	1583	63	19	40	94
S15	2000	333	35	7	27	97
S16	1150	192	24	3	21	100
S17	1150	192	30	12	17	97
S18	1350	225	25	6	17	92
