# Supplementary material for: Cystoisospora suis Control in Europe Is Not Always Effective
Source: Front Vet Sci. 2020 Mar 4;7:113. doi: 10.3389/fvets.2020.00113 (PMC7064465; doi:10.3389/fvets.2020.00113)
Supplement: Supplementary file 2 [file Data_Sheet_1.pdf]

Questionnaire for piglet samples

Farmer's name and address:

Information reg. the farm

Number of sows:

Most used breed:

Number of other pigs:

Breeding only

Closed system

Information on management

All-in-all-out yes/no

Cleaning

Disinfection, disinfectant

Treatment in the first three weeks of life:

Toltrazuril: day of treatment, product

Iron: day of treatment

Antibiotics:

Information on sample(s)

Litter/sow number:

Date of sampling:

Birth date of litter:

Litter size (day of sampling):

Diarrhoea yes/no: colour, consistency, onset

health observations: unthriftiness, high mortality

other observations
